# Predictive values of tumor necrosis factor-α for depression treatment outcomes: effect modification by hazardous alcohol consumption

**DOI:** 10.1038/s41398-021-01581-7

**Published:** 2021-09-02

**Authors:** Wonsuk Choi, Hee-Ju Kang, Ju-Wan Kim, Hee Kyung Kim, Ho-Cheol Kang, Ju-Yeon Lee, Sung-Wan Kim, Robert Stewart, Jae-Min Kim

**Affiliations:** 1 Department of Internal Medicine, Chonnam National University Hwasun Hospital, Chonnam National University Medical School, Hwasun, Korea; 2 Department of Psychiatry, Chonnam National University Medical School, Gwangju, Korea; 3 Institute of Psychiatry, Psychology and Neuroscience, King’s College London, London, UK; 4 South London and Maudsley NHS Foundation Trust, London, UK

**Keywords:** Depression, Predictive markers

## Abstract

Inflammation is potentially associated with poor antidepressant treatment outcomes. Pro-inflammatory cytokines are influenced by hazardous alcohol consumption. The aim of the present study was to investigate the effects of the serum tumor necrosis factor-α (sTNF-α) level on antidepressant treatment outcomes in terms of the 12-week and 12-month remission rates and 24-month relapse rate, and to investigate the potential modifying effects of alcohol consumption on these associations in patients with depressive disorders. At baseline, sTNF-α was measured and alcohol-related data from the Alcohol Use Disorders Identification Test (AUDIT) and consumption history were collected from 1094 patients. Patients received stepwise antidepressant treatment. Remission at 12 weeks and 12 months was defined as a Hamilton Depression Rating Scale (HAMD) score ≤ 7. Relapse (HAMD score ≥ 14) was identified until 24 months for those who had initially responded (HAMD score <14) at 12 weeks. Higher sTNF-α levels were found to have significant effects on the 12-week and 12-month non-remission and 24-month relapse rates. These effects were more prominent in those with low levels of alcohol consumption (AUDIT score ≤ 8 or no current alcohol consumption); the effects were not significant in those exhibiting hazardous alcohol consumption (AUDIT score > 8 or current drinking). Significant interactions were found for the 12-month non-remission and relapse rates, although the interaction was not statistically significant for 12-week remission. In conclusion, baseline sTNF-α levels may be a useful predictor for both short- and long-term antidepressant treatment outcomes, and the consideration of alcohol consumption status may increase predictability, in particular for long-term outcomes.

## Introduction

Inflammation is considered an important contributor to the pathophysiology of depressive disorders [1, 2]. Depression risk is bidirectionally associated with the levels of pro-inflammatory cytokines such as tumor necrosis factor-α (TNF-α), interleukin-1β (IL-1β), and IL-6 [3–6]. Moreover, treatment-resistant depressed patients had greater inflammation than responders in recent studies [7–9]. Despite the importance of verifying the longitudinal association between pro-inflammatory cytokines and depression treatment outcomes, as predictors of differential responses to treatment, the results of such research have been inconsistent. Some studies have reported that high pro-inflammatory cytokine levels were associated with worse treatment outcomes [10–14], whereas others have found an association with better treatment outcomes [15, 16] or no significant association [17].

Chronic alcohol consumption has been associated with an increase in pro-inflammatory cytokines in various tissues including the blood, liver, and brain [18]. Pro-inflammatory cytokines including TNF-α, IL-1, and IL-6 were found to increase in individuals exhibiting chronic alcohol consumption. Increased circulating pro-inflammatory cytokines were significantly associated with parameters related to liver injury, hepatic protein synthesis, and serum immunoglobulin concentrations [19]. Moreover, alcohol consumption was associated with increased expression of pro-inflammatory cytokines due to nuclear factor-κB activation in the brain [20, 21]. Given the association between alcohol consumption and increased pro-inflammatory cytokines, alcohol consumption may modify the association between pro-inflammatory cytokines and antidepressant treatment outcomes. However, this has not been studied so far.

Using data from a prospective study of Korean patients with depressive disorders receiving stepwise antidepressant treatment, we investigated the effects of the baseline serum TNF-α (sTNF-α) level on antidepressant treatment outcomes including 12-week and 12-month remission and 24-month relapse. In addition, we investigated the potential modifying effects of alcohol consumption on the association between baseline sTNF-α level and treatment outcome in patients with depressive disorders.

## Materials and methods

### Study outline

This study was conducted as a component of the MAKE Biomarker discovery for Enhancing antidepressant Treatment Effect and Response (MAKE BETTER) program. Details of the study have been reported as a design paper [22] and the study is registered at cris.nih.go.kr (identifier: KCT0001332). To reflect real-world treatment settings, participants were enrolled independent of depression subtypes or physical comorbidity. Treatment interventions were also carried out in a naturalistic manner in terms of determining the type, dose, and regimen of antidepressant and other medications, which were based on patient preference as well as clinician decisions, although they were guided by pre-planned measurements and time points. Details of the overall treatment steps and strategies in this study have been published elsewhere [23]. A summary of the stepwise pharmacotherapy administered in this study is shown in the Supplementary Methods. After 3 weeks of antidepressant monotherapy, the next treatment steps following alternative strategies could be started every 3 weeks during the acute treatment phase (3, 6, 9, and 12 weeks) and every 3 months during the continuation (6, 9, and 12 months) and maintenance (15, 18, 21, and 24 months) treatment phases. For those who responded in the 12-week acute treatment phase, the relapse status was evaluated from 6 to 24 months. All data on socio-demographic and clinical characteristics at baseline and treatment-related variables at follow-up were recorded using a structured clinical report form (CRF) by clinical research coordinators who were blind to treatment strategies. These staff were trained in CRF record-taking and data collection methods by the research psychiatrists. Each patient’s data were recorded on a CRF, uploaded to the MAKE BETTER study website (http://icreat.nih.go.kr/icreat/webapps/com/hismainweb/jsp/cdc_n2.live) within 3 days, and were monitored by data management personnel at the research center. This study was approved by the Chonnam National University Hospital Institutional Review Board (CNUH 2012-014).

### Participants

Among patients who had visited the outpatient psychiatric department of Chonnam National University Hospital, those with depressive disorders, who satisfied the eligibility criteria (Supplementary Methods), were consecutively recruited from March 2012 to April 2017. All inclusion instances represented new treatment episodes, i.e., taking newly initiated antidepressant treatment, whether depressive symptoms were first onset or recurrent. As the primary objective of the MAKE BETTER study was to discover predictive markers for antidepressant treatment outcomes, all participants received, with their consent, antidepressant-based treatment only.

### Exposure variables

#### sTNF-α level

Participants were instructed to fast (except water) overnight prior to blood sampling. Participants were then asked to sit quietly and relax for 25–45 min before blood samples were drawn. The sTNF-α level was measured using a Human TNF-α Quantikine HS ELISA HSTA00D system (R&D Systems, MN, USA) at the Global Clinical Central Lab (Yongin, Korea). Patients were divided according to sTNF-α level into low- and high-sTNF-α groups based on the median value in the main analysis. In addition, the sTNF-α level was analyzed as a continuous variable in subsequent analyses.

#### Alcohol consumption

Self-reported assessment of alcohol use, drinking patterns, and alcohol-related issues was performed at baseline using the Alcohol Use Disorders Identification Test (AUDIT) scale [24]. Patients were divided into two groups based on their AUDIT scores: those with scores below 8 (AUDIT score < 8) and those with scores of 8 or higher (AUDIT score ≥ 8), which is the criterion for distinguishing between hazardous alcohol use and low-risk consumption according to World Health Organization (WHO) guidelines. Patients were categorized according to their alcohol drinking status at baseline as non-current drinkers, including never drinkers and ex-drinkers, or current drinkers.

### Baseline covariates

The socio-demographic characteristics collected included age, sex, years of formal education, marital status (currently married or not), cohabitation status (living alone or not), religion (religious observance vs. none), occupation (currently employed or not), monthly income (above or below 2000 USD), and body mass index (BMI). Serum biomarkers for hepatic damage, including aspartate aminotransferase (AST) and alanine aminotransferase (ALT), were assessed. The clinical characteristics evaluated comprised diagnoses of depressive disorders as mentioned above with certain specifiers, i.e., age at onset, duration of illness, history of previous depressive episodes (recurrent or first episode), number of previous depressive episodes, duration of present episode, family history of depression, history of suicide attempts, and number of concurrent physical disorders (determined using a questionnaire enquiring about 15 different systems or disorders). Assessment scales for investigating symptoms and functions were administered. Depressive symptoms were evaluated using the Hamilton Depression Rating Scale (HAMD) [25], anxiety symptoms using the Hospital Anxiety Depression Scale-anxiety subscale (HADS-A) [26], quality of life using the EuroQol-5D instrument (EQ-5D) [27], and level of functioning using the Social and Occupational Functioning Assessment Scale.

### Outcome measures

#### Remission

Remission was defined as a HAMD score ≤ 7. Remission was evaluated at 12 weeks and 12 months, to allow comparisons with other studies that used 6- to 12-month follow-ups [28, 29]. In addition, cases of loss to follow-up or failure to take antidepressants were considerable after 1 year to estimate accurate remission rates.

#### Relapse

Patients who responded during the 12-week acute treatment phase (HAMD score < 14) were included for relapse analyses. For re-assessment, relapse status was examined using the same protocol starting at 12 weeks and every 3 months thereafter up to 24 months. Relapse was defined as a HAMD score ≥ 14, consistent with previous studies [29, 30].

### Statistical analysis

Patients’ baseline data were compared based on their sTNF-α level (low vs. high), AUDIT score (<8 vs. ≥8), and alcohol drinking status (current non-drinker vs. current drinker) using independent *t*-tests or *χ*^2^-tests. The effects of the sTNF-α level (binary variable [low vs. high] in the main analysis; continuous variable in additional analyses) on the 12-week remission, 12-month remission, and 24-month relapse rates were analyzed using logistic regression before and after adjusting for potential covariates. Then, the modifying effects of alcohol consumption (AUDIT score [<8 vs. ≥8] in the main analysis and alcohol drinking status [current non-drinker vs. current drinker] in the additional analyses) on the associations were estimated using models with the same adjustments. All statistical tests were two-sided and *P*-values < 0.05 were considered to indicate statistical significance. Statistical analyses were performed using IBM SPSS Statistics (version 25).

## Results

### Recruitment and flow

**Fig. 1:**
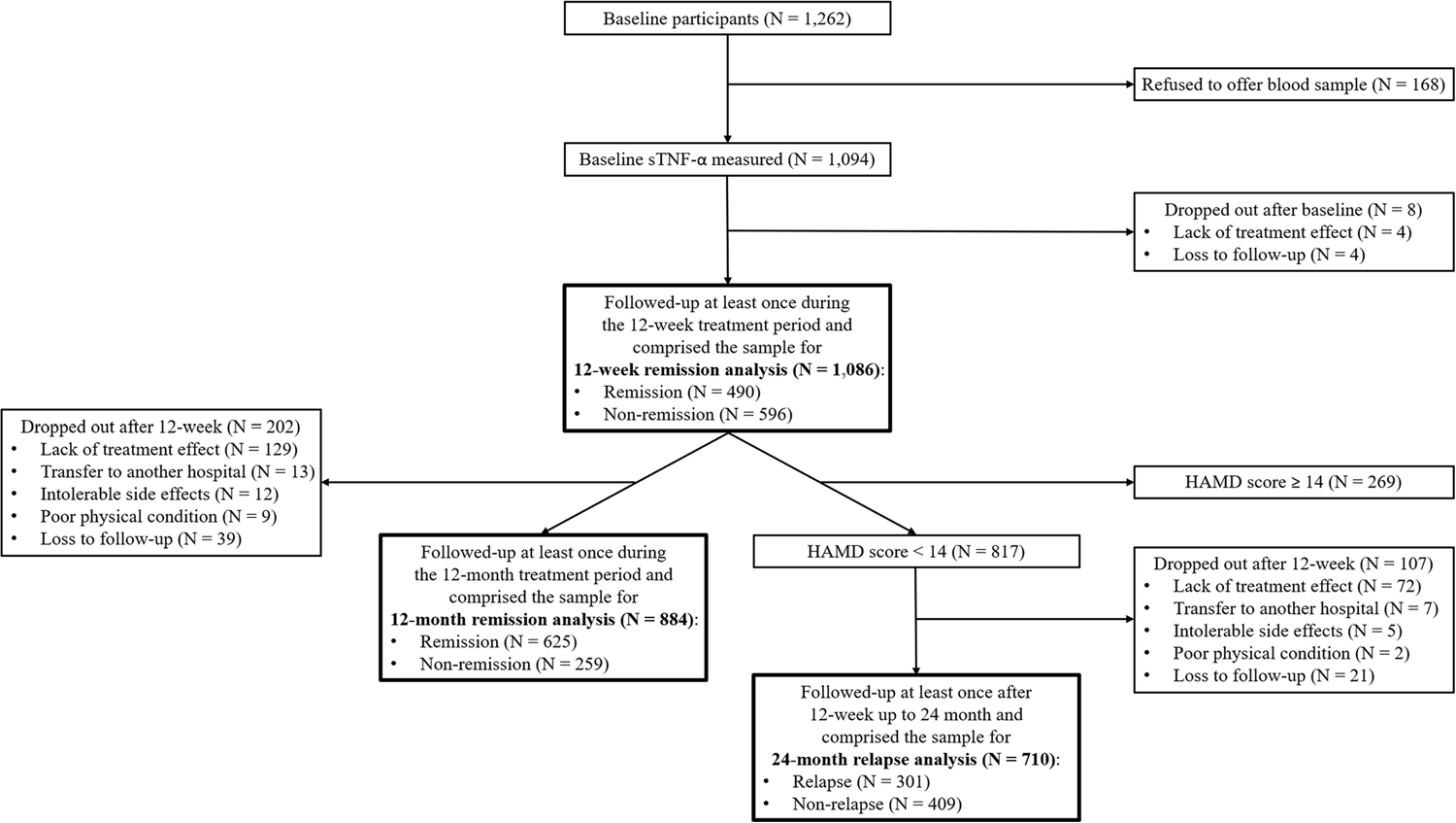
Patient recruitment and flow. sTNF-α, serum tumor necrosis factor-α; HAMD, Hamilton Depression Rating Scale.

Patient recruitment and flow are described in Fig. 1. Among 1262 patients evaluated at baseline, sTNF-α levels were measured in 1094 (86.7%) and 1086 (86.1%) were followed up at least once during the 12-week treatment period. Reasons for dropout included a lack of treatment effect (*N* = 4) and loss to follow-up (*N* = 4). There were no statistical differences in any baseline characteristics between the 1094 participants included and the 8 not followed. Of those included, 490 (45.1%) scored ≤7 on the HAMD at the 12-week assessment point. After the acute treatment phase, 884 (81.4%) patients were followed up at least once until the 12-month follow-up. Reasons for dropout included a lack of treatment effect (*N* = 129), transfer to another hospital (*N* = 13), intolerable side effects (*N* = 12), poor physical condition (*N* = 9), and loss to follow-up (*N* = 39). Dropout at 12 months was significantly associated with an unemployment status, a higher rate of melancholic features, and a higher EQ-5D score at baseline. However, dropout at 12 months was not associated with the sTNF-α level, AUDIT score, or alcohol drinking status. Of 884 patients, 625 (70.7%) scored ≤7 on the HAMD at the 12-month assessment point. The 24-month relapse analysis showed that of 817 patients who scored below 14 on the HAMD at the 12-week assessment point, 710 (86.9%) were evaluated at least once during the 24-month follow-up period after the acute treatment phase. Reasons for dropout at this stage included a lack of treatment effect (*N* = 72), transfer to another hospital (*N* = 7), intolerable side effects (*N* = 5), poor physical condition (*N* = 2), and loss to follow-up (*N* = 21). Dropout at 24 months was significantly associated with a shorter duration of the present episode and a lower rate of melancholic features. However, dropout at 24 months was not associated with the sTNF-α level, AUDIT score, or alcohol drinking status. Of 710 patients, 301 (42.4 %) scored ≥14 on the HAMD at the 24-month assessment point.

### Baseline characteristics by exposure level

Baseline characteristics stratified by sTNF-α level in patients who underwent up to 12 weeks of treatment (acute treatment phase) are summarized in Table 1. A high sTNF-α level was significantly associated with an older age, male sex, a lower education level, monthly income < 2000 USD, higher BMI, older age at onset, longer duration of the present episode, a suicide attempt history, and higher AST and ALT levels. In addition, baseline characteristics were compared according to AUDIT score and alcohol drinking status (Supplementary Tables 1 and 2, respectively). AUDIT scores ≥ 8 and current drinking were both significantly associated with a younger age, male sex, a higher education level, an unmarried status, a non-religious status, monthly income not <2000 USD, the presence of atypical features, lower age at onset, higher number of depressive episodes, a suicide attempt history, and higher AST and ALT levels. Based on the statistical significance (*P* < 0.05) and potential multicollinearity, 13 variables (sex, education, marital status, religious observance, monthly income, melancholic features, atypical features, age at onset, number of depressive episodes, duration of present episode, history of suicide attempts, HADS-A score, and initial antidepressant type) were included in the subsequent adjusted analyses. The baseline sTNF-α level was higher in patients with AUDIT scores ≥ 8 compared to those with AUDIT scores < 8 and in current drinkers compared to current non-drinkers.

**Table 1:** Baseline characteristics according to serum tumor necrosis factor-α (sTNF-α) level in patients with depressive disorders, who underwent 12 weeks of treatment (*N* = 1086).

	Low sTNF-α (*N* = 542)	High sTNF-α (*N* = 544)	Statistical coefficients^a^	*P*-value
Socio-demographic characteristics				
Age, mean (SD) years	54.9 (14.6)	59.0 (14.9)	*t* = −4.565	*P* < 0.001
Sex, *N* (%) female	395 (72.9)	350 (64.3)	*χ*^2^ = 9.192	*P* = 0.002
Education, mean (SD) years	9.6 (4.5)	8.6 (5.0)	*t* = 3.437	*P* = 0.001
Marital status, *N* (%) unmarried	174 (32.1)	152 (27.9)	*χ*^2^ = 2.239	*P* = 0.135
Living alone, *N* (%)	89 (16.4)	78 (14.3)	*χ*^2^ = 0.905	*P* = 0.342
Religious observance, *N* (%)	299 (55.2)	308 (56.6)	*χ*^2^ = 0.232	*P* = 0.630
Unemployed status, *N* (%)	152 (28.0)	164 (30.1)	*χ*^2^ = 0.582	*P* = 0.446
Monthly income, *N* (%) <2000 USD	299 (55.2)	349 (64.2)	*χ*^2^ = 9.115	*P* = 0.003
Clinical characteristics				
Body mass index, mean (SD) kg/m^2^	22.9 (3.2)	23.5 (3.1)	*t* = −3.127	*P* = 0.002
Major depressive disorder, *N* (%)	458 (84.5)	467 (85.8)	*χ*^2^ = 0.388	*P* = 0.533
Melancholic feature, *N* (%)	76 (14.0)	86 (15.8)	*χ*^2^ = 0.683	*P* = 0.409
Atypical feature, *N* (%)	41 (7.6)	28 (5.1)	*χ*^2^ = 2.667	*P* = 0.102
Age at onset, mean (SD) years	50.1 (16.0)	53.6 (17.2)	*t* = −3.515	*P* < 0.001
Duration of illness, mean (SD) years	4.8 (8.1)	5.3 (9.8)	*t* = −0.992	*P* = 0.321
Recurrent depression, *N* (%)	284 (52.4)	286 (52.6)	*χ*^2^ = 0.003	*P* = 0.954
Number of depressive episodes, mean (SD)	1.1 (1.5)	1.1 (1.4)	*t* = 0.168	*P* = 0.867
Duration of present episode, mean (SD) months	6.5 (9.5)	8.3 (11.2)	*t* = −2.786	*P* = 0.005
Family history of depression, *N* (%)	79 (14.6)	79 (14.5)	*χ*^2^ = 0.001	*P* = 0.980
History of suicide attempt, *N* (%)	36 (6.6)	59 (10.8)	*χ*^2^ = 6.010	*P* = 0.014
Assessment scales, mean (SD) scores				
Hamilton Depression Rating Scale	20.9 (4.1)	20.6 (4.2)	*t* = 0.998	*P* = 0.319
Hospital Anxiety & Depression Scale-anxiety subscale	11.9 (4.0)	11.7 (4.1)	*t* = 1.100	*P* = 0.272
EuroQol-5D	8.9 (1.3)	8.9 (1.7)	*t* = −0.465	*P* = 0.642
Social and Occupational Functional Assessment Scale	55.8 (7.1)	56.1 (7.9)	*t* = −0.643	*P* = 0.520
Laboratory tests, median (IQR) U/L				
Aspartate aminotransferase	22.0 (8.0)	23.0 (10.0)	*U* = 129262.0	*P* < 0.001
Alanine aminotransferase	17.0 (10.0)	19.0 (12.0)	*U* = 134185.5	*P* = 0.010

^a^Independent two-sample *t*-test or *χ*^2^-test, as appropriate.

### Effects of the sTNF-α level and alcohol consumption on treatment outcomes

The effects of the sTNF-α level on 12-week remission, 12-month remission, and 24-month relapse are shown in Table 2. A higher sTNF-α level, considered as both a binary and continuous variable, was associated with 12-week non-remission, 12-month non-remission, and 24-month relapse in both unadjusted and adjusted analyses. However, the AUDIT score and alcohol drinking status were not significantly associated with 12-week remission, 12-month remission, or 24-month relapse (Supplementary Table 3).

**Table 2:** Effects of serum tumor necrosis factor-α level as a binary and continuous variable on the incidence of 12-week and 12-month remission and 24-month relapse.

Exposure	Group	12-Week remission (*N* = 1086)				12-Month remission (*N* = 884)				24-Month relapse (*N* = 710)			
		*N*	No. (%) presence	OR (95% CI)		*N*	No. (%) presence	OR (95% CI)		*N*	No. (%) presence	OR (95% CI)	
				Unadjusted	Adjusted^a^			Unadjusted	Adjusted^a^			Unadjusted	Adjusted^a^
sTNF-α (binary)	Low	542	261 (48.2)	Reference	Reference	434	329 (75.8)	Reference	Reference	344	112 (32.6)	Reference	Reference
	High	544	229 (42.1)	0.78 (0.62–0.99)^*^	0.77 (0.60–1.00)^*^	450	296 (65.8)	0.61 (0.46–0.82)^**^	0.61 (0.45–0.83)^**^	366	189 (51.6)	2.21 (1.63–3.00)^***^	2.47 (1.78–3.43)^***^
sTNF-α (increasing)	NA	1086	NA	0.75 (0.58–0.97)^*^	0.74 (0.57–0.97)^*^	884	NA	0.55 (0.41–0.76)^**^	0.56 (0.41–0.78)^***^	710	NA	1.49 (1.06–2.09)^*^	1.56 (1.10–2.21)^*^

*sTNF-α* serum tumor necrosis factor-α.

^a^Adjusted for sex, education, marital status, religious observance status, monthly income, presence of melancholic features, presence of atypical features, age at onset, number of depressive episodes, duration of present episode, history of suicide attempts, HADS-A score, and initial antidepressant type.

*P < 0.05.

**P < 0.01.

***P < 0.001.

**Fig. 2:**
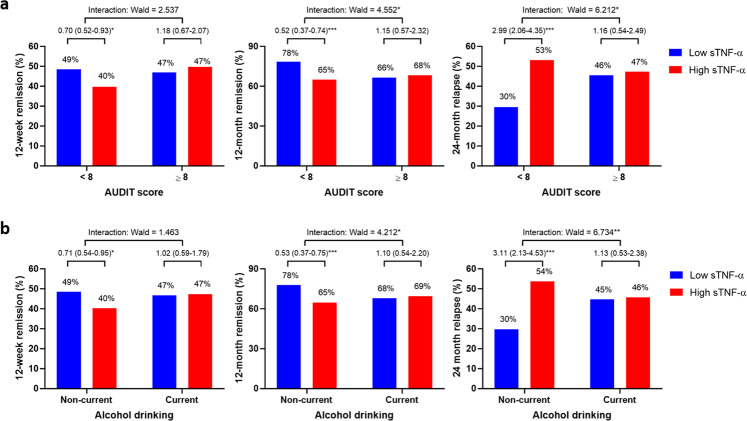
Effects of the interaction between the serum tumor necrosis factor-α level and alcohol consumption on the incidence of 12-week and 12-month remission and 24-month relapse in patients with depressive disorders. Effects of the interaction between the serum tumor necrosis factor-α (sTNF-α) level and the Alcohol Use Disorders Identification Test (AUDIT) score (**a**) or alcohol drinking status (**b**) on the incidence of 12-week and 12-month remission and 24-month relapse in patients with depressive disorders. Data are Wald’s scores after adjustment for sex, education, marital status, religious observance status, monthly income, the presence of melancholic features, the presence of atypical features, age at onset, number of depressive episodes, duration of present episode, history of suicide attempts, HADS-A score, and initial antidepressant type. *P < 0.05, **P < 0.01 and ***P < 0.001.

The modifying effects of alcohol consumption on the associations of the sTNF-α level with 12-week remission, 12-month remission, and 24-month relapse are shown in Fig. 2. High sTNF-α levels were prominently and significantly associated with all three treatment outcomes among those with AUDIT scores < 8 and among current non-drinkers, whereas the association was not significant in patients with AUDIT scores ≥ 8 or in current drinkers. In addition, the interaction between sTNF-α and alcohol consumption status had significant effects on 12-month non-remission and 24-month relapse, but not on 12-week non-remission, after adjustment for relevant covariates. The results were similar regardless of whether sTNF-α was analyzed as a continuous or binary variable.

## Discussion

In the present study, using data from a naturalistic prospective study that reflected real-world clinical practice, we identified significant effects of higher sTNF-α levels on 12-week non-remission, 12-month non-remission, and 24-month relapse. Moreover, these effects were modified by the effects of hazardous alcohol consumption (AUDIT score > 8 or current drinking) on the baseline sTNF-α level. In particular, a high sTNF-α level at baseline in patients with low levels of alcohol consumption (AUDIT score < 8 or current non-drinker status) was a significant predictor of long-term antidepressant treatment outcomes such as non-remission at 12 months and relapse at 24 months. These findings were robust after adjustment for relevant covariates. Although the incidence of 12-week non-remission was higher among patients with high sTNF-α levels combined with AUDIT scores < 8 or a current non-drinker status, the interaction terms were not statistically significant.

As noted in the “Introduction”, the association between high levels of baseline pro-inflammatory cytokines, such as TNF-α, IL-1β, and IL-6, and antidepressant treatment outcomes has been inconsistent in previous studies [10–17] despite widespread confidence in the role of inflammatory signaling in the pathogenesis of depression [1, 2]. However, focusing only on TNF-α, a higher peripheral level [10] and higher expression levels in circulating leukocytes [13, 14] predicted a worse antidepressant treatment response. In addition, plasma TNF levels were positively correlated with the number of failed treatment trials in unmedicated, medically stable patients with major depressive disorders [11]. Thus, our finding that higher TNF-α levels predict a poor antidepressant treatment outcome is in line with previous studies.

Taking one step beyond previous studies that evaluated short-term antidepressant treatment responses (up to 12 weeks) [10, 13, 14], we demonstrated the role of higher TNF-α levels as a biomarker for 12-month non-remission and 24-month relapse in patients with depressive disorders. To the best of our knowledge, this is the first study to show that baseline sTNF-α can influence long-term antidepressant treatment outcomes. Considering that recently published clinical guidelines recommend maintaining antidepressants for up to 9 months [31, 32] and our study is a naturalistic prospective study using a diverse range of treatment strategies, it would be worthwhile to further evaluate baseline sTNF-α as a predictor of long-term antidepressant treatment outcomes in real-world clinical settings.

There are several mechanisms by which higher TNF-α levels may contribute to worse antidepressant treatment outcomes. First, TNF-a signaling has been suggested to decrease serotonin availability by increasing serotonin reuptake through upregulation of the expression and function of serotonin transporters [33]. Therefore, TNF-a signaling may mitigate the efficacy of selective serotonin reuptake inhibitors, which are the most commonly used antidepressants. Second, TNF-α signaling has been shown to inhibit brain-derived neurotrophic factor (BDNF) and block neurogenesis [34]. As BDNF fosters neurogenesis, which is an important prerequisite for an antidepressant response, upregulated TNF-α signaling may reduce antidepressant efficacy through the alteration of BDNF pathways. Third, preclinical studies have shown that TNF-α signaling induces upregulated extra-synaptic glutamate receptor signaling by stimulating astrocytes to increase the release of glutamate, while decreasing the expression of glutamate transporters responsible for glutamate uptake [35]. Increased extra-synaptic glutamate receptor signaling can contribute to decreased levels of BDNF and excitotoxicity, thereby decreasing the efficacy of antidepressants.

Hazardous alcohol consumption modified the association between the baseline sTNF-α level and antidepressant treatment outcomes, although this had no direct effect on treatment outcomes. 12-week and 12-month non-remission and 24-month relapse were more frequently observed in patients with high TNF-α levels, who had AUDIT scores < 8 or were not current drinkers compared to those with AUDIT score ≥ 8 or current drinkers. As in depression [1], chronic alcohol consumption increases pro-inflammatory cytokines in both the periphery and the central nervous system [19–21]. Therefore, high sTNF-α levels may not predict future antidepressant treatment outcomes in alcoholic patients as a result of the elevation of sTNF-α due to alcohol consumption. These results suggest that the sTNF-α level can be used as a biomarker for predicting antidepressant treatment outcomes, particularly in those with low alcohol consumption, but caution in interpretation may be needed for patients exhibiting hazardous alcohol consumption.

A previous study using the AUDIT to examine hazardous alcohol consumption in 1100 Korean subjects suggested a cutoff score of 11 based on drinking quantity and 8 based on the CAGE [36]. As one of the two cutoff scores for Koreans, and the WHO cutoff score, was 8, an AUDIT score ≥ 8 was defined as hazardous alcohol consumption in this study.

Interestingly, a significant interaction between sTNF-α level and alcohol consumption was observed only for long-term outcomes (incidence of 12-month remission and 24-month relapse). These results are likely because patients whose alcohol consumption at baseline was classified as hazardous probably continued to consume more alcohol during the 2-year study period compared with patients with low levels of alcohol consumption. Differences in absolute alcohol consumption between the two groups might have increased during long-term follow-up, resulting in differences in the interaction terms. However, as we did not investigate the subjects’ alcohol consumption status during the study period, further research is needed.

The strengths of this study include the large sample size and the long follow-up period. Participants were evaluated using a structured research protocol and standardized scales. This is the first study to examine the effects of TNF-α level on long-term antidepressant treatment outcomes and the modification of this association via hazardous alcohol consumption. Furthermore, as this was a naturalistic prospective study that reflected actual clinical situations, the results obtained in this study can serve as a basis for developing biomarkers for antidepressant treatment outcomes in real-world clinical practice.

Several limitations to this study should be borne in mind when considering inferences. First, we were not able to assess the association between treatment-related changes in sTNF-α and treatment outcomes, as longitudinal data on sTNF-α levels were lacking. Given that systemic pro-inflammatory signaling is considered an important pathological process in depression, treatment-related changes in sTNF-α may be associated with treatment outcomes. Second, because of the naturalistic design of the study, treatment was determined based on patient preference with a physician’s guidance rather than using a preset protocol; thus, inter-physician variability might have affected the treatment outcomes. However, as physicians guided treatment decisions without knowing the baseline sTNF-α level, it is unlikely that inter-physician variability affected the outcomes. Third, due to the heterogeneity of prescribed antidepressants, it is difficult to determine the differential predictive effects of sTNF-α on antidepressant treatment outcomes according to antidepressant type. However, as our results were derived after adjusting for initial antidepressant type, the predictive effect of sTNF-α on antidepressant treatment outcomes is more likely to be a generalized conclusion, independent of the type of treatment regimen. Fourth, continuation and maintenance treatment phase follow-up rates were relatively low compared to that for the acute treatment phase. Given the distinct characteristics of participants who were lost to follow-up in continuation (higher rate of unemployment, higher rate of melancholic features, and higher EQ-5D scores) and maintenance treatment phase (shorter present-episode duration and lower rate of melancholic features), the results might have been affected. However, this possibility is unlikely, because baseline sTNF-α levels, AUDIT scores, and the alcohol consumption status did not differ according to follow-up status during the continuation and maintenance treatment phase. Fifth, as our patients were from a single-center, the generalizability of our findings may be limited.

In conclusion, a high sTNF-α level predicted 12-week non-remission, 12-month non-remission, and 24-month relapse in patients with depressive disorders. In addition, hazardous alcohol consumption modified the association between high sTNF-α levels and long-term antidepressant treatment outcomes. The results suggest that when using the sTNF-α level as a predictor of long-term antidepressant treatment outcomes, the consideration of hazardous alcohol consumption may increase predictability. With regard to therapeutic considerations, special attention is needed for patients who have high sTNF-α levels but do not exhibit hazardous alcohol consumption; however, further studies are needed to evaluate whether adjunctive anti-inflammatory treatments may be beneficial in this subpopulation.

## Supplementary information

Supplementary Table 1, 2, and 3
