# Supplementary material for: Predictive values of tumor necrosis factor-α for depression treatment outcomes: effect modification by hazardous alcohol consumption
Source: Transl Psychiatry. 2021 Sep 2;11:450. doi: 10.1038/s41398-021-01581-7 (PMC8413287; doi:10.1038/s41398-021-01581-7)
Supplement: Supplementary file 1 — Supplementary Table 1, 2, and 3 [file 41398_2021_1581_MOESM1_ESM.docx]

**Supplementary Information**

**Predictive values of tumor necrosis factor-alpha for depression treatment outcomes: effect modification by hazardous alcohol consumption**

Wonsuk Choi^a^, Hee-Ju Kang^b^, Ju-Wan Kim^b^, Hee Kyung Kim^a^, Ho-Cheol Kang^a^, Ju-Yeon Lee^b^, Sung-Wan Kim^b^, Robert Stewart^c,d^, and Jae-Min Kim^b^

^a^Department of Internal Medicine, Chonnam National University Hwasun Hospital, Chonnam National University Medical School, Hwasun, Korea, ^b^Department of Psychiatry, Chonnam National University Medical School, Gwangju, Korea, ^c^King’s College London, Institute of Psychiatry, Psychology and Neuroscience, London, UK, ^d^South London and Maudsley NHS Foundation Trust, London, UK.

**Supplementary Methods**

**Stepwise pharmacotherapy**

Before treatment commencement, a comprehensive review was made of each patient’s clinical manifestation (e.g. presence of psychotic or anxiety symptoms), severity of illness, physical comorbidity and medication profile, and history of previous treatments. Minimal and maximal dosages of pharmacological agents were determined considering existing treatment guidelines ^1,2^. In the first treatment Step 1, patients received antidepressant monotherapy, taking into consideration these data and treatment guidelines ^2-4^ for 3 weeks. Antidepressants used were bupropion, desvenlafaxine, duloxetine, escitalopram, fluoxetine, mirtazapine, paroxetine, sertraline, venlafaxine, and vortioxetine. After Step 1 antidepressant monotherapy, next step pharmacotherapy could be administered every 3 weeks during the acute treatment phase and every 3 months during the continuation and maintenance treatment phase, whenever needed. At the end of each step, overall effectiveness and tolerability were reviewed before proceeding with measurement-based next-step treatments. In cases of insufficient improvement (a HAMD score reduction of < 30% from the previous step) or intolerable side effects, patients were instructed to choose whether they would prefer to remain in the current step or enter next-step strategies with switching to other antidepressants (S), augmentation with drugs other than antidepressants (A), combination with other antidepressants (C), or multiple strategies (S+A; S+C; A+C; S+A+C). Patients were also allowed to receive next step treatment if they showed sufficient improvement (a HAMD score reduction of ≥ 30% from the previous step) and absent/tolerable side effects. For determining treatment strategies, each patient’s preference was given priority to maximize medication compliance and treatment outcomes. Antidepressants switched or combined were bupropion, desvenlafaxine, duloxetine, escitalopram, fluoxetine, mirtazapine, paroxetine, sertraline, venlafaxine, and vortioxetine. Augmentation drugs were buspirone, lithium, triiodothyronine, and atypical antipsychotics including aripiprazole, risperidone, olanzapine, quetiapine, and ziprasidone. Since the number entered into Step 5 or above was small, treatment steps were classified into Step 1, 2, 3, and 4 (including Step 5+) in the analysis. Medication adherence was estimated based on the tablet counts at every visit and was defined as poor in cases with less than 50% intake ^5^.

**Eligibility criteria**

Because the purpose of the study was to reflect the real-world clinical environment, broad inclusion and minimal exclusion criteria were adopted. Inclusion criteria were: i) aged older than 7 years; ii) diagnosed with major depressive disorder (MDD), dysthymic disorder, or depressive disorder not otherwise specified (NOS), using the Mini-International Neuropsychiatric Interview (MINI) ^6^, a structured diagnostic psychiatric interview based on the Diagnostic and Statistical Manual of Mental Disorders, Fourth Edition (DSM-IV) criteria; iii) Hamilton Depression Rating Scale (HAMD) ^7^ score ≥ 14; iv) able to complete questionnaires, understand the objective of the study, and sign the informed consent form. Exclusion criteria were as follows: i) unstable or uncontrolled medical condition; ii) unable to complete the psychiatric assessment or comply with the medication regimen, due to a severe physical illness; iii) current or lifetime DSM-IV diagnosis of bipolar disorder, schizophrenia, schizoaffective disorder, schizophreniform disorder, psychotic disorder NOS, or other psychotic disorder; iv) history of organic psychosis, epilepsy, or seizure disorder; v) history of anticonvulsant treatment; vi) hospitalization for any psychiatric diagnosis apart from depressive disorder (e.g., alcohol/drug dependence); vii) electroconvulsive therapy received for the current depressive episode; viii) pregnant or breastfeeding. All participants reviewed the consent form and written informed consent was obtained. For participants aged under 16, written consent was obtained from a parent or legal guardian, and written assent was obtained from the participant.

**References**

1 Anderson, I. M. et al. Evidence-based guidelines for treating depressive disorders with antidepressants: a revision of the 2000 British Association for Psychopharmacology guidelines. *J Psychopharmacol* **22**: 343-396 (2008).

2 Bauer, M. et al. World Federation of Societies of Biological Psychiatry (WFSBP) guidelines for biological treatment of unipolar depressive disorders, part 1: update 2013 on the acute and continuation treatment of unipolar depressive disorders. *World J Biol Psychiatry* **14**: 334-385 (2013).

3 Malhi, G. S. et al. Royal Australian and New Zealand College of Psychiatrists clinical practice guidelines for mood disorders. *Aust N Z J Psychiatry* **49**: 1087-1206 (2015).

4 Kennedy, S. H. et al. Canadian Network for Mood and Anxiety Treatments (CANMAT) 2016 Clinical Guidelines for the Management of Adults with Major Depressive Disorder: Section 3. Pharmacological Treatments. *Can J Psychiatry* **61**: 540-560 (2016).

5 Haynes, R. B., McDonald, H. P. & Garg, A. X. Helping patients follow prescribed treatment: clinical applications. *JAMA* **288**: 2880-2883 (2002).

6 Sheehan, D. V. et al. The Mini-International Neuropsychiatric Interview (M.I.N.I.): the development and validation of a structured diagnostic psychiatric interview for DSM-IV and ICD-10. *J Clin Psychiatry* **59 Suppl 20**: 22-33;quiz 34-57 (1998).

7 Hamilton, M. A rating scale for depression. *J Neurol Neurosurg Psychiatry* **23**: 56-62 (1960).

| **Supplementary Table 1**  Baseline characteristics according to the Alcohol Use Disorder Identification Test (AUDIT) score in patients with depressive disorders who underwent 12-weeks of treatment (N = 1,086) | | | | | | | |  |
| --- | --- | --- | --- | --- | --- | --- | --- | --- |
|  | |  | | **Up to 12-week treatment (N = 1086)** | | | | |
|  |  | | AUDIT score < 8 (N = 842) | | AUDIT score ≥ 8 (N = 244) | Statistical coefficients^a^ | P-value |  |
| **Socio-demographic characteristics** |  | |  | |  |  |  |  |
| Age, mean (SD) years |  | | 59.3 (13.5) | | 48.7 (16.4) | t = 9.275 | P < 0.001 |  |
| Sex, N (%) female |  | | 652 (77.4) | | 93 (38.1) | χ^2^ = 135.783 | P < 0.001 |  |
| Education, mean (SD) years |  | | 8.5 (4.8) | | 11.3 (4.2) | t = -8.975 | P < 0.001 |  |
| Marital status, N (%) unmarried |  | | 238 (28.3) | | 88 (36.1) | χ^2^ = 5.478 | P = 0.019 |  |
| Living alone, N (%) |  | | 132 (15.7) | | 35 (14.3) | χ^2^ = 0.258 | P = 0.611 |  |
| Religious observance, N (%) |  | | 510 (60.6) | | 97 (39.8) | χ^2^ = 33.251 | P < 0.001 |  |
| Unemployed status, N (%) |  | | 254 (30.2) | | 62 (25.4) | χ^2^ = 2.075 | P = 0.150 |  |
| Monthly income, N (%) <2,000 USD |  | | 538 (63.9) | | 110 (45.1) | χ^2^ = 27.824 | P < 0.001 |  |
| **Clinical characteristics** |  | |  | |  |  |  |  |
| Body mass index, mean (SD) kg/m^2^ |  | | 23.2 (3.1) | | 23.4 (3.3) | t = -0.939 | P = 0.348 |  |
| Major depressive disorder, N (%) |  | | 717 (85.2) | | 208 (85.2) | χ^2^ = 0.001 | P = 0.972 |  |
| Melancholic feature, N (%) |  | | 135 (16.0) | | 27 (11.1) | χ^2^ = 3.678 | P = 0.055 |  |
| Atypical feature, N (%) |  | | 40 (4.8) | | 29 (11.8) | χ^2^ = 16.185 | P < 0.001 |  |
| Age at onset, mean (SD) years |  | | 54.1 (15.6) | | 44.1 (17.9) | t = 7.878 | P < 0.001 |  |
| Duration of illness, mean (SD) years |  | | 5.2 (9.4) | | 4.6 (7.5) | t = 1.158 | P = 0.248 |  |
| Recurrent depression, N (%) |  | | 430 (51.1) | | 140 (57.4) | χ^2^ = 3.019 | P = 0.082 |  |
| Number of depressive episodes, mean (SD) |  | | 1.0 (1.4) | | 1.3 (1.7) | t = -2.863 | P = 0.004 |  |
| Duration of present episode, mean (SD) months |  | | 7.4 (10.6) | | 7.5 (9.6) | t = -0.163 | P = 0.871 |  |
| Family history of depression, N (%) |  | | 117 (13.9) | | 41 (16.8) | χ^2^ = 1.287 | P = 0.257 |  |
| History of suicide attempt, N (%) |  | | 46 (5.5) | | 49 (20.1) | χ^2^ = 50.647 | P < 0.001 |  |
| **Assessment scales, mean (SD) scores** |  | |  | |  |  |  |  |
| Hamilton Depression Rating Scale |  | | 20.8 (4.1) | | 20.4 (4.2) | t = 1.329 | P = 0.184 |  |
| Hospital Anxiety & Depression Scale-anxiety subscale |  | | 11.7 (4.0) | | 12.1 (4.1) | t = -1.464 | P = 0.144 |  |
| EuroQol-5D |  | | 9.0 (1.5) | | 8.7 (1.5) | t = 2.481 | P = 0.013 |  |
| Social and Occupational Functional Assessment Scale |  | | 55.8 (7.4) | | 56.4 (7.6) | t = -1.080 | P = 0.281 |  |
| **Laboratory tests, median (IQR) U/L** |  | |  | |  |  |  |  |
| Aspartate aminotransferase |  | | 22.0 (9.0) | | 24.0 (10.0) | U = 90115.5 | P = 0.003 |  |
| Alanine aminotransferase |  | | 17.0 (10.0) | | 20.5 (12.8) | U = 85329.5 | P < 0.001 |  |
| Tumor necrosis factor-alpha |  | | 0.59 (0.41) | | 0.62 (0.41) | U = 91670.5 | P = 0.010 |  |

^a^Independent two sample t-test or χ^2^ test, as appropriate.

| **Supplementary Table 2**  Baseline characteristics according to the alcohol drinking status in patients with depressive disorders who underwent 12-weeks of treatment (N = 1,086) | | | | | |
| --- | --- | --- | --- | --- | --- |
|  |  | Non-current drinkers (N = 840) | Current drinkers (N = 246) | Statistical coefficients^a^ | P-value |
| **Socio-demographic characteristics** |  |  |  |  |  |
| Age, mean (SD) years |  | 59.3 (13.6) | 48.8 (16.3) | t = 9.216 | P < 0.001 |
| Sex, N (%) female |  | 649 (77.3) | 96 (39.0) | χ^2^ = 129.155 | P < 0.001 |
| Education, mean (SD) years |  | 8.5 (4.8) | 11.2 (4.3) | t = -8.568 | P < 0.001 |
| Marital status, N (%) unmarried |  | 237 (28.2) | 89 (36.2) | χ^2^ = 5.746 | P = 0.017 |
| Living alone, N (%) |  | 132 (15.7) | 35 (14.2) | χ^2^ = 0.323 | P = 0.570 |
| Religious observance, N (%) |  | 506 (60.2) | 101 (41.1) | χ^2^ = 28.397 | P < 0.001 |
| Unemployed status, N (%) |  | 253 (30.1) | 63 (25.6) | χ^2^ = 1.875 | P = 0.171 |
| Monthly income, N (%) <2,000 USD |  | 534 (63.6) | 114 (46.3) | χ^2^ = 23.473 | P < 0.001 |
| **Clinical characteristics** |  |  |  |  |  |
| Body mass index, mean (SD) kg/m^2^ |  | 23.2 (3.1) | 23.4 (3.3) | t = -1.160 | P = 0.246 |
| Major depressive disorder, N (%) |  | 716 (85.2) | 209 (85.0) | χ^2^ = 0.012 | P = 0.914 |
| Melancholic feature, N (%) |  | 136 (16.2) | 26 (10.6) | χ^2^ = 4.737 | P = 0.030 |
| Atypical feature, N (%) |  | 40 (4.8) | 29 (11.8) | χ^2^ = 15.790 | P < 0.001 |
| Age at onset, mean (SD) years |  | 54.1 (15.7) | 44.3 (17.7) | t = 7.855 | P < 0.001 |
| Duration of illness, mean (SD) years |  | 5.2 (9.4) | 4.5 (7.7) | t = 1.159 | P = 0.247 |
| Recurrent depression, N (%) |  | 432 (51.4) | 138 (56.1) | χ^2^ = 1.663 | P = 0.197 |
| Number of depressive episodes, mean (SD) |  | 1.0 (1.4) | 1.3 (1.7) | t = -2.341 | P = 0.020 |
| Duration of present episode, mean (SD) months |  | 7.4 (10.6) | 7.4 (9.5) | t = 0.074 | P = 0.941 |
| Family history of depression, N (%) |  | 119 (14.2) | 39 (15.9) | χ^2^ = 0.436 | P = 0.509 |
| History of suicide attempt, N (%) |  | 48 (5.7) | 47 (19.1) | χ^2^ = 42.746 | P < 0.001 |
| **Assessment scales, mean (SD) scores** |  |  |  |  |  |
| Hamilton Depression Rating Scale |  | 20.8 (4.1) | 20.5 (4.2) | t = 0.914 | P = 0.361 |
| Hospital Anxiety & Depression Scale-anxiety subscale |  | 11.7 (4.0) | 12.3 (4.1) | t = -2.078 | P = 0.038 |
| EuroQol-5D |  | 9.0 (1.5) | 8.8 (1.6) | t = 1.285 | P = 0.200 |
| Social and Occupational Functional Assessment Scale |  | 55.9 (7.4) | 56.1 (7.7) | t = -0.378 | P = 0.705 |
| **Laboratory tests, median (IQR) U/L** |  |  |  |  |  |
| Aspartate aminotransferase |  | 22.0 (9.0) | 24.0 (10.0) | U = 90536.0 | P = 0.003 |
| Alanine aminotransferase |  | 17.0 (10.0) | 21.0 (13.0) | U = 85591.5 | P < 0.001 |
| Tumor necrosis factor-alpha |  | 0.59 (0.41) | 0.63 (0.40) | U = 90427.5 | P = 0.003 |

^a^Independent two sample t-test or χ^2^ test, as appropriate.

| **Supplementary Table 3**  Effects of the alcohol use disorder identification test (AUDIT) score and alcohol drinking status on the incidence of 12-week and 12-month remission and 24-month relapse | | | | | | | | | | | | | |
| --- | --- | --- | --- | --- | --- | --- | --- | --- | --- | --- | --- | --- | --- |
| Exposure | Group | **12-week remission (N = 1,086)** | | | | **12-month remission (N = 884)** | | | | **24-month relapse (N = 710)** | | | |
|  |  | N | No. (%) presence | OR (95% CI) | | N | No. (%) presence | OR (95% CI) | | N | No. (%) presence | OR (95% CI) | |
|  |  |  |  | Unadjusted | Adjusted^a^ |  |  | Unadjusted | Adjusted^a^ |  |  | Unadjusted | Adjusted^a^ |
| AUDIT | < 8 | 842 | 372 (44.2) | Reference | Reference | 688 | 493 (71.7) | Reference | Reference | 553 | 228 (41.2) | Reference | Reference |
|  | ≥ 8 | 244 | 118 (48.4) | 1.18 (0.89-1.57) | 1.32 (0.95-1.84) | 196 | 132 (67.3) | 0.92 (0.58-1.15) | 0.90 (0.60-1.35) | 157 | 73 (46.5) | 1.24 (0.87-1.77) | 1.19 (0.78-1.82) |
|  |  |  |  |  |  |  |  |  |  |  |  |  |  |
| Alcohol drinking | Non-current | 840 | 374 (44.5) | Reference | Reference | 689 | 491 (71.3) | Reference | Reference | 553 | 230 (41.6) | Reference | Reference |
|  | Current | 246 | 116 (47.2) | 1.11 (0.84-1.48) | 1.23 (0.88-1.70) | 195 | 134 (68.7) | 0.89 (0.63-1.25) | 0.99 (0.66-1.47) | 157 | 71 (45.2) | 1.16 (0.81-1.66) | 1.09 (0.71-1.65) |

^a^Adjusted for sex, education, marital status, religious observance status, monthly income, presence of melancholic features, presence of atypical features, age at onset, number of depressive episodes, duration of present episode, history of suicide attempts, HADS-A score, and initial antidepressant type.
